# Screening of Sensitive Items on Gross Motor Development in Toddlers Aged 24~36 Months and Analysis of the Factors Influencing the Passing Rate

**DOI:** 10.3390/children7110226

**Published:** 2020-11-13

**Authors:** Deng Chen, Jinming Yu, Jiwei Wang, Yue Bai, Yaxuan Zhang, Xinyuan Lu, Beibei Che, Sikun Chen, Yilu Huang, Xiaoguang Yang

**Affiliations:** Key Laboratory of Public Health Safety, Ministry of Education, National Health Commission Key Laboratory of Health Technology Assessment (Fudan University), School of Public Health, Fudan University, Shanghai 200433, China; 19111020021@fudan.edu.cn (D.C.); jmy@fudan.edu.cn (J.Y.); jiweiwang@fudan.edu.cn (J.W.); 19211020074@fudan.edu.cn (Y.B.); 18111020002@fudan.edu.cn (Y.Z.); 18211020007@fudan.edu.cn (X.L.); 18211020047@fudan.edu.cn (B.C.); skchen20@fudan.edu.cn (S.C.); 20211020036@fudan.edu.cn (Y.H.)

**Keywords:** sensitive items, gross motor, toddlers, influencing factors

## Abstract

Background: To save assessment time and improve the efficiency, it is necessary to find sensitive indicators from the test items of gross motor development in the C-LAP system for children aged 24~36 months and analyze the influencing factors of the passing rate of these indicators. Methods: This retrospective study was conducted among 1354 toddlers (3058 person-times) aged 24 to 36 months in Beijing, Shanghai, Guangdong between January 2013 and December 2019. A linear regression model and Cox regression model were performed to screen sensitive indicators and explore their influencing factors, respectively. Results: “Walk backwards”, “Stand from supine position” and “Hop with one foot at least twice” are the three sensitive indicators for evaluating the development of gross motor function in 24~36 month old children. The child’s physiological age at first measurement and region are the two common independent factors influencing the passing rate of the three items, while paternal age and education may influence one or two of them. Conclusions: “Walk backwards”, “Stand from supine position” and “Hop with one foot at least twice” are sensitive indicators for the effective evaluation of the development of gross motor function in 24~36 month old children, and their passing rates are influenced by some demographic variables.

## 1. Introduction

Motor development is a process experienced by a child from birth up to approximately six or seven years of age, the most favorable stage being when stimulating each one of the capacities that comprise their fundamental components [1]. As an important part of motor development, gross motor development involves the muscles of the arms, legs, feet or the whole body, taking the forms of heading up, turning over, crawling, sitting, rolling, standing, walking, running and jumping, etc. Studies have shown that children’s gross motor development plays an important role in children’s physical and mental health [2,3], self-confidence and self-esteem building [4], and even academic performance [5]. In addition, it is strongly correlated with other areas of children’s neuropsychological development, including fine motor skills, self-help ability, cognition, language and social ability [6,7].

In recent years, the prevalence of various neuropsychological developmental diseases among children in China has become an increasingly prominent public health problem. A meta-analysis showed that the total prevalence of autism spectrum disorder (ASD) in Chinese children from 2000 to 2016 was 0.24% (male: 0.35%, female: 0.07%; urban: 0.17%, rural: 0.09%) [8]. After screening children with ASD in Jilin Province, Shenzhen City and Jiamusi City, a study found that the prevalence of ASD in Chinese children was close to that in western countries, at around 1% [9]. From 2001 to 2018, the epidemiological survey results indicate that the prevalence of cerebral palsy (CP) varied between 1.29‰ and 5.06‰ in all regions of China [10,11,12]. The total prevalence ratio of Down Syndrome (DS) was reported to be 3.05 per 10,000 births between 2003 and 2011, and the proportion of DS diagnosed prenatally increased from 7.55% between 1996 and 2002 to 47.70% between 2003 and 2011 [13]. Due to these diseases, developmental disorders or delaying of gross motor function in children was too common to be mere happenstance, such as restricted and repetitive behaviors in children with ASD [14], uncontrolled movements in children with CP [15], and delaying of standing position and walking ability in children with DS [16].

It is well known that timely detection of developmental delays and disorders, especially in the motor domain, is very important for early intervention during the first few years of life. The timing of movement delays and their specificity to the corresponding disease are critical; for instance, a study suggested that differences in motor function may contribute to early identification of autism [17]. In an influential and widely publicized study, autism’s movement disorders were found to exist at birth and can be appropriately used to diagnose autism in the first few months of life [18]. Similar movement difficulties were observed in a later study looking at early detection of Asperger’s syndrome, including asymmetries in prone lying and crawling, log rolling, asymmetrical tonic neck reflex (ATNR) that persisted past the age of developmental appropriateness, and a lack of protective responses when balance was lost [19].

Without any doubt, a toddler needs to take a giant leap in motor development before becoming a child. Motor development is usually described by motor milestones [20], while many systematic evaluation scales, such as C-LAP, have gained popularity among experts in this field recently. The standardized procedure for some evaluation requires each child to be demonstrated by an evaluator, followed by practice and acting trials [21]. Some pervasively used assessment tools provided meaningful measurements starting at 2 years old [22]; however, these measurements often took at least half to an hour to complete, which may be impractical for screening purposes. Especially for the C-LAP system with six areas, if each area takes at least half an hour, the overall time would be too long. Even if parents agree to let their children take the test, it is not easy for the young subjects being tested to maintain sustained attention. Based on these findings, researchers and clinicians are eager to test fewer items to further reduce the time involved in each evaluation [23].

Many studies improved and simplified the existing complicated assessment systems with qualitative or quantitative methods, to achieve the purpose of increasing efficiency and revenue. For example, item screening was conducted through the application of multi-step measurement, which included the generation, reduction, and scale creation of items, development of test manuals, and pilot evaluation of the clinical feasibility of each item [24]. Another technology named Rasch analysis has also been used to develop new measures and test existing ones [25], which was based on a probabilistic model using maximum likelihood estimation to sort of items and the subjects at the same time. In addition, there are other methods to extract assessments subsets, and the evaluation results of the subsets sorted out by these methods have been proved to be in good agreement with those obtained by applying the original evaluation system [26,27].

Therefore, in this paper, by screening discriminative items from the gross motor developmental test system of C-LAP, we tried to find sensitive indicators that could quickly and effectively qualitatively distinguish the degree of motor development in children aged 24~36 months. In this way, we can improve the detection efficiency of motor development disorders in children aged 24~36 months, set some specific, proper parenting goals for parents and teachers, and even enable parents to have a general idea of the motor development of their children at home without special testing. Meanwhile, survival analysis was performed to observe the time needed to pass the test of the target items in the children who failed to pass them during the first test. We also adopted Cox regression analysis to identify the potential factors influencing the outcome and passing time of the test for these three items.

## 2. Materials and Methods

### 2.1. Participants

This retrospective study was carried out in Shanghai VIP Health Care Co., Ltd. (C-LAP, Shanghai, China). We collected the longitudinal testing data of gross motor development of the toddlers aged 24 to 36 months in Beijing, Shanghai, Guangdong between January 2013 and December 2019. A total of 1354 cases (3058 person-times) were included in our study, of which 419 were tested at least twice, while the others were tested only once. The written informed consent to participate was obtained from the parent or legal guardian of the children, for whom some parenting advice was provided.

### 2.2. Measurement

Chinese Learning Accomplishment Profile (C-LAP) system was applied to evaluate the development of gross motor function, the reliability (internal consistency) of which was supported by Cronbach’s α coefficient of 0.81, and the correlation coefficient between physiological age and developmental age of gross motor tested was 0.95 [28]. After controlling for the physiological monthly age, the partial correlation coefficient between developmental monthly ages measured in different dimensions was lower than 0.70, indicating a good construct validity of the evaluation system [28].

The context of the instrument is arranged from easy to difficult, and only children that pass the former questions are able to answer the latter ones. The first item corresponding to the physiological month age was selected as the starting point of the test; if a child passes it, then the next item will be tested, and if not, the researcher goes back 8 items from it and reaches at a new starting point, and so on.

### 2.3. Screening of Items

For children aged 24 to 36 months, the C-LAP system contains 7 items: “Walk in a straight line”, “Jump down a step”, “Walk backward”, “Stand up from supine position”, “Change feet to climb stairs”, “Balance on one foot” and “Hop with one foot at least twice”. However, if a child aged 24~36 months fails any of these items, the items for 23 or even lower months of age will be tested. As a matter of convenience, we selected the 7 items above as candidate items, which were ranked by the passing rate (difficulty), and then the items with larger contribution rate were selected through the linear regression model. Combining results of the two steps above, we eventually determined the sensitive indicators with better difficulty gradient and higher contribution, which were further verified by survival analysis.

### 2.4. Statistical Analysis

Descriptive data were presented as percentages (CI). A linear regression model and Cox regression model were performed to screen sensitive indicators and explore their associated factors, respectively. Log-rank, Two-stage, and Restricted Mean Survival Time (RMST) tests were adopted to compare the difference between two survival curves with or without crossing. SAS 9.4 and R 3.6.2 statistical software packages were used for data analysis and plotting. In cases where some values were missing, we assumed that the data were missing at random. A P value of less than 0.05 was considered as the significant level.

## 3. Results

### 3.1. Basic Demographic Information

**Figure 1:**
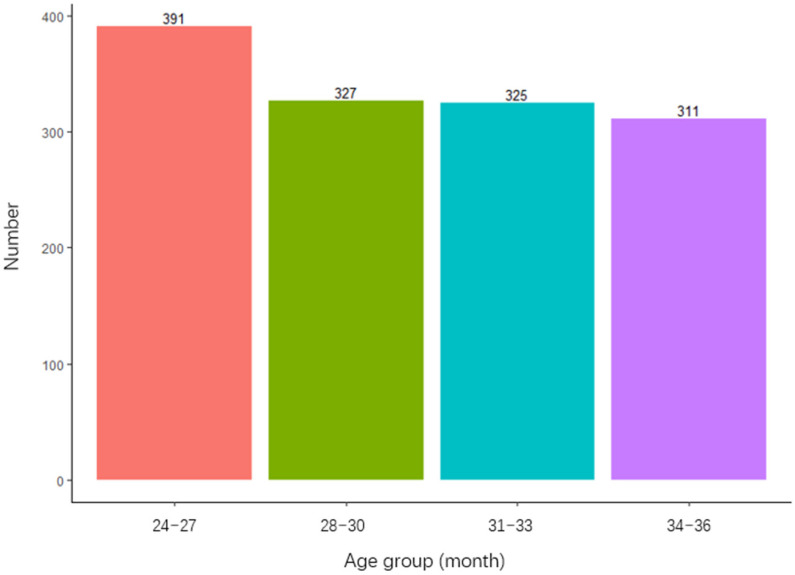
Age distribution of 1354 toddlers at first assessment.

In this study, a total of 1354 toddlers (3058 person-times) from 24 months to 36 months in Beijing, Shanghai and Guangdong were collected, among whom 222 cases (16.4%) were in Beijing, 898 (66.3%) in Shanghai and 234 (17.3%) in Guangdong. The subjects included 743 (54.9%) boys and 610 (45.1%) girls. Overall, 869 (64.2%) of the fathers were over 30 years old, while 693 (51.2%) of the mothers were over 30 years old. A total of 919 (67.9%) fathers held a bachelor’s degree or above, and 917 (67.8%) mothers held a bachelor’s degree or above. The physiological age of all children at first measurement is shown in the Figure 1, and the overall distribution was relatively balanced, among which the number of children aged 24 to 27 monthsold accounted for the largest proportion (28.9%).

### 3.2. Screening of the Main Sensitive Indicators of Gross Motor Development

Among 1354 children (if there were repeated measurements, only the first measurement results were counted), the passing rate of the seven items in all physiological age groups were shown in Table 1. The toddlers performed better in the items of “Walking in a straight line” and “Jumping down a step”, with a passing rate of 84.3% (95% CI: 82.3~86.2%) and 73.3% (95% CI: 70.9~75.7%), respectively. Starting with the item “Walk backward”, the passing rate decreased by a larger margin of 54.8% (95% CI: 52.1~57.5%), and the passing rate of the remaining items reduced successively, until it came down to the last one, “Hop with one foot at least twice”, the passing rate of which was only 13.0% (95% CI: 11.3~14.9%). These passing rates in each sub-age group and the whole age group effectively reflect the difficulty gradient of the items and provide a reference for us to select the appropriate outcome indicators.

To find sensitive or representative indicators among the seven items, we constructed a linear model, taking the scores of 1354 children’s gross motor function in the system as the dependent variable and the seven items above as the independent variables. Although all items were statistically significant in univariate linear regression with positive coefficients, the coefficient sign of the item “Balance on one foot “ became negative in multivariate regression and each item still had statistical significance in the model (*p* < 0.001) (Table 2). In the test of multicollinearity, it was found that the variance inflation factor (VIF) of each item was lower than 2, and the pairwise correlation coefficient between items was lower than 0.53.

All seven items were prioritized, and Table 3 showed the ranking results. Among them, “Stand up from supine position”, “Walk backwards”, “Change feet to climb stairs” and “Hop with one foot at least twice” were the four indicators with higher weight coefficients. The other three items were considered to be omitted on account of their weight coefficients that were lower than 10. Due to the close differences (about 20%) in the passing rates between “Stand up from supine position” and “Walk backwards” and between “Hop with one foot at least twice” and “Stand up from supine position” (Table 1), the three items “Walk backwards”, “Stand up from supine position” and “Hop with one foot at least twice” were reserved eventually. The item “Change feet to climb stairs” was excluded in that the weight coefficient of it was much lower than that of the “Stand up from supine position” (Table 3) and its inability to form uniform difficulty differences with other three items. Empirically, we can refer to the method of normal assessment (passing rate in this paper) and qualitatively divide children who pass the items “Walk backwards”, “Stand up from supine position”, and “Hop with one foot at least twice” into three grades, such as “Not bad”, “Good” and “Very good”, respectively (Table 4).

### 3.3. Survival Analysis

Survival analysis was carried out with the three identified final items above as outcome indicators and observation time as survival time. Toddlers aged 24 to 33 months who failed at first measurement were selected for prospective observation, and the test results of each item at the end of observation were assigned respectively, in which “passed” was assigned 1 and “failed” was assigned 0.

**Figure 2:**
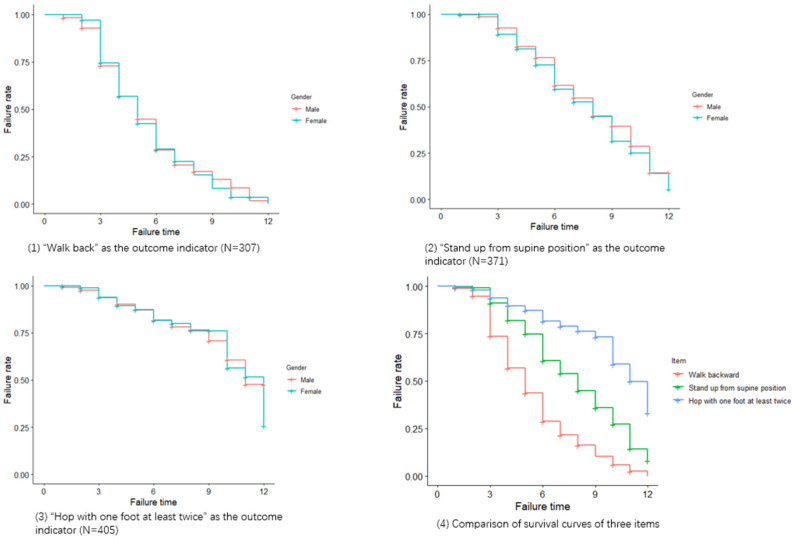
Survival curves of the three items.

Figure 2 shows the survival curve of the three items. The survival curve test indicated that there was no significant difference between different genders in the overall survival curve of each item (*p* > 0.05). The median “failure time” of the items “Walk backwards”, “Stand from supine position” and “Hop with one foot at least twice” was 5 months (95% CI: 5~6 months), 8 months (95% CI: 7~9 months) and 11 months (95% CI: 10~12 months), respectively, and there was statistically significant difference between the three survival curves by pairwise test (*p* < 0.05/3). The failure rate of the three evaluation grades at each time point is provided (Table 5), and also presented a gradient change, indicating that the selected indicators were effective enough to distinguish the development of gross motor function in children aged 24~36 months.

### 3.4. Cox Regression Analysis

Since the sample size of survival analysis with the outcome indicators “Walk backwards” (N = 307), “Stand from supine position” (N = 371) and “Hop with one foot at least twice” (N = 405) was sufficient (more than 20 times the number of variables), all of the variables except for gender (child’s physiological age at first measurement, paternal age and education, maternal age and education, Region) were included as the independent variable. Cox regression models were constructed by taking the test results and observation time of the three indicators as dependent variables respectively, using the methods of stepwise regression. Cox regression results are shown in Table 6, which demonstrated that the child’s physiological age at first measurement and region were the two common independent influencing factors. The passing rate of the three outcome indicators increased with the child’s physiological age. Taking children in Beijing as a reference, children in Guangdong had a higher pass rate in “Walk backwards” and “Hop with one foot at least twice”, while children from Shanghai had a higher passing rate in “Hop with one foot at least twice” and a lower passing rate in “Stand from supine position”. Compared with children whose father was more than 30 years old, children with a father ≤30 years old had higher passing rates for “Walk backwards” and “Hop with one foot at least twice”, and children whose father held a bachelor’s degree or above had a higher passing rate in the program of “Stand from supine position” than those with a father with a lower degree.

## 4. Discussion

Referring to the model of the E-LAP system in the USA [29], the C-LAP system adopted some items from the classic scales and arranged these items into different groups of month age according to their order from easy to difficult, so as to achieve a combination of standard reference and normative assessment. Our system is mainly aimed at the preliminary screening of general childhood development problems, and its reliability and validity have been supported by long-term data collected from Shanghai, Beijing and Guangdong in China. We selected items from this system that are suitable for the qualitative assessment of gross motor development of children aged 24~36 months, taking into account not only the representativeness of these classic items, but also rapid assessment of children’s development of gross motor, as well as goals appropriately set for children’s motor development.

To our knowledge, this study is the first to screen the sensitive indicators of the motor development of children aged 24~36 months and evaluate the passing rate and the time it takes using survival analysis. The principal finding of this study is that “Walk backwards”, “Stand from supine position” and “Hop with one foot at least twice” are the sensitive indicators for the effective evaluation of the development of gross motor function in 24~36 month old children. The results of survival analysis show that these three indicators make a good distinction for children’s gross motor development, in that the median passing time of them for children who failed at the first measurement present a gradient change (5, 8, and 11 months).

Appropriate coordination of children is important not only for their overall development, but also for their health, psychosocial development, academic performance and so on [30]. “Walk backward” measures the balance of infants and young children, and previous research showed that the test system of gross motion coordination of Körperkoordinationstest für Kinder (KTK) including the sub-test “backward walking” had high application value [31]. In particular, backward walking highlights prominent gait asymmetries in children with hemiplegia and diplegia from cerebral palsy and can give a more comprehensive assessment of gait pathology. The observed spatiotemporal asymmetry assessments may reflect both impaired supraspinal control and impaired state of the spinal circuitry [32]. Children with cerebellar lesions in a study showed significant performance decrements in all tasks compared with the controls, particularly in the movement coordination test and paced stepping task [33]. Some therapists have long advocated backward walking on treadmills or the ground in clinical practice to treat patients in rehabilitation for sports or functional mobility/gait [34]. This was confirmed by a preliminary study which suggested that reverse treadmill therapy could improve the walking ability of children with spastic cerebral palsy [35]. Another study also found that backward walking training is more effective than forward walking training on spatiotemporal gait parameters, and gross motor function measures in children with hemiparetic cerebral palsy [36]. One possible explanation is that backward walking uses the same rhythm circuitry of forward walking, but additionally requires specialized control circuits [37].

Getting off the floor is an important milestone in a child’s development. Clinicians should evaluate children’s ability to rise from a supine position to an erect stance and encourage children to improve this ability or, when it is lacking, acquire it [38]. There have been many studies on the movement patterns of children from supine to upright position, and the main conclusion is that younger children tend to use asymmetrical movement patterns such as the involvement of full or partial rotation of the trunk until the trunk reaches either a prone position or a side-facing position, while older children tend to use symmetrical movement patterns, such as sitting up from the supine position and then moving their “shoulders forward to gain a quadrupedal position” [39,40]. In conclusion, multisegmental analysis has been used to describe systematically movement patterns of children standing up from a supine position [41]. Studies demonstrated that children with spastic diplegia or hemiplegic cerebral palsy stand up from a supine position mostly using general patterns of movement described in normal toddlers and children, but they showed a markedly reduced intra- and inter-individual variability compared to normal age-matched controls [38,39]. The results of another study indicate that the children with Development Delay (DD) followed the typical developmental sequences, but with different maturation speeds and greater variability [42]. All these show that “Stand from supine position” can be used as a valuable identification indicator of early gross motor developmental disorder in children, and has been widely used in the screening or research of neuropsychological developmental disorder in children [43].

Hopping requires skillful organization of the spatial–temporal relationship between body segments [44] and adequate timing and coordination [45]. Ghanem introduced hopping on one foot as the best test used to assess the strength of the triceps surae muscle in children over 5 years of age [46]. Holm, Tveter et al. believed that the ‘‘hop on one leg’’ test can discriminate between well-coordinated and less-coordinated children aged 7~12 years. In a study evaluating the effectiveness of Taekwondo interventions in children with ASD, the one leg hop test turned out to be a very important indicator [47], although the evaluation has not yet been verified widely [48].

Our observations, combined with those of other investigators, suggest that physiological age at first measurement of children aged 24~36 months is a protective factor for the passing rate of various indicators [38,39]. Our study suggests that gender has no significant effect on the passing rate of the three indicators, which is in line with the result from prior studies in which hardly any performance differences between males and females in the items “Walk backwards” and “Hop with one foot at least twice” were observed [49,50]. However, it represents a departure from the existing literatures, in which girls were more skillful than boys in hopping and scored better on the balance task, while boys performed better on the strength-oriented task [44,51,52].

In our study, fathers aged ≥30 years is a risk factor for children passing the items “Walk backwards” and “Hop with one foot at least twice”. Previous studies have also suggested that a child whose father has older childbearing age will be at greater risk for ASD and impaired development of gross motor [53]. As it was found that father’s education background has a positive influence on children’s gross motor development in another study, we found father’s education of “less than a bachelor’s degree” may be a risk factor for “Stand from supine position” [54]. Children in different regions also have significant differences in the passing rate of each item, which agrees well with the conclusions from prior studies that there are regional differences in the development of gross motor function [55,56].

The present study has two main strengths. First, the large sample size and long sample collection timespan can make the calculation results of the passing rate of different indicators more stable and the conclusion more reliable and convincing. Second, some subjects were visited several times, and the longitudinal data obtained are powerful to prospectively analyze the test results and follow-up time of various indicators, revealing the rule of development and influence factors of gross motor in different individuals on the timeline.

Several limitations should be considered when interpreting our findings. First, the data in this paper are historical and retrospective, and the accumulation process was not strictly controlled, which hinder us from collecting some other relatively important indicators that have an impact on the results. Second, the research objects in this study are mainly collected from relatively developed provinces and cities in Mainland China, such as Beijing, Shanghai, Guangdong, etc., hence the generalization of the results is limited. In further study, the data of other regions will be collected and analyzed. Third, parents’ coping styles may vary to some certain extent after children failed the first test, which may have an impact on the follow-up outcomes. However, in general, this effect can be small, as all parents were provided with some parenting advice and guidance.

## Figures and Tables

**Table 1:** Passing number (N) and rate (%) of each item in different age groups.

Item	Passing Number (Total)	Passing Rate (Total) (95% CI)	Passing Number (34~36 Months Age)	Passing Rate (34~36 Months Age) (95% CI)	Passing Number (31~33 Months Age)	Passing Rate (31~33 Months Age) (95% CI)	Passing Number (28~30 Months Age)	Passing Rate (28~30 Months Age) (95% CI)	Passing Number (24~27 Months Age)	Passing Rate (24~27 Months Age) (95% CI)
Walk in a straight line	1142	84.3 (82.3, 86.2)	288	92.6 (89.1, 95.3)	306	94.2 (91.0, 96.4)	285	87.2 (83.0, 90.6)	263	67.3 (62.4, 71.9)
Jump down a step	993	73.3 (70.9, 75.7)	282	90.7 (86.9, 93.7)	278	85.5 (81.2, 89.2)	248	75.8 (70.8, 80.4)	185	47.3 (42.3, 52.4)
Walk backward	742	54.8 (52.1, 57.5)	239	76.8 (71.8, 81.4)	241	74.2 (69.0, 78.8)	186	56.9 (51.3, 62.3)	76	19.4 (15.6, 23.7)
Stand up from supine position	477	35.2 (32.7, 37.8)	215	69.1 (63.7, 74.2)	160	49.2 (43.7, 54.8)	76	23.2 (18.8, 28.2)	26	6.6 (4.4, 9.6)
Change feet to climb stairs	353	26.1 (23.7, 28.5)	160	51.4 (45.7, 57.1)	109	33.5 (28.4, 39.0)	65	19.9 (15.7, 24.6)	19	4.9 (3.0, 7.5)
Balance on one foot	196	14.5 (12.6, 16.5)	101	32.5 (27.3, 38.0)	59	18.2 (14.1, 22.8)	27	8.3 (5.5, 11.8)	9	2.3 (1.1, 4.3)
Hop with one foot at least twice	176	13.0 (11.3, 14.9)	89	28.6 (23.7, 34.0)	47	14.5 (10.8, 18.8)	31	9.5 (6.5, 13.2)	9	2.3 (1.1, 4.3)

**Table 2:** Linear regression model used to determine the significance of the influence of each item on the developmental age tested of gross motor function.

	β Coefficient (95% CI)	Std. Error	*t* Value	*p* Value
Intercept	22.08 (21.78, 22.38)	0.15	143.20	<0.001
Walk in a straight line	0.91 (0.54, 1.28)	0.19	4.85	<0.001
Jump down a step	1.56 (1.25, 1.88)	0.16	9.61	<0.001
Walk backward	2.71 (2.42, 3)	0.15	18.60	<0.001
Stand up from supine position	3.47 (3.18, 3.75)	0.15	23.91	<0.001
Change feet to climb stairs	2.97 (2.65, 3.3)	0.17	17.77	<0.001
Balance on one foot	−1.63 (−2.04, −1.21)	0.21	−7.70	<0.001
Hop with one foot at least twice	3.29 (2.86, 3.71)	0.22	15.27	<0.001

**Table 3:** Order of importance of 7 items.

Item	Weight Coefficient
Stand up from supine position	23.91
Walk backward	18.60
Change feet to climb stairs	17.77
Hop with one foot at least twice	15.27
Jump down a step	9.61
Balance on one foot	7.70
Walk in a straight line	4.85

**Table 4:** The sensitive indicators determined eventually and the corresponding evaluation result if some item is passed.

Item	Evaluation Result
Walk backward	Not bad
Stand up from supine position	Good
Hop with one foot at least twice	Very good

**Table 5:** Failure rate at each time point.

Item	Time Point (Month)	Number of Subjects That May Pass	Number of Passing	Failure Rate	Std. Error	95% CI
Walk backward	1	307	3	0.99	0.01	(0.98, 1)
	3	278	75	0.74	0.03	(0.69, 0.79)
	6	96	108	0.29	0.03	(0.23, 0.35)
	9	25	32	0.10	0.02	(0.07, 0.16)
	12	2	13	0		
Stand up from supine position	1	371	1	1	0	(0.99, 1)
	3	340	30	0.91	0.02	(0.88, 0.94)
	6	159	73	0.61	0.03	(0.55, 0.67)
	9	60	37	0.36	0.04	(0.29, 0.44)
	12	7	21	0.08	0.03	(0.04, 0.19)
Hop with one foot at least twice	1	405	2	1	0	(0.99, 1)
	3	367	22	0.94	0.01	(0.91, 0.96)
	6	169	29	0.82	0.02	(0.77, 0.86)
	9	74	10	0.73	0.03	(0.67, 0.80)
	12	12	18	0.33	0.08	(0.21, 0.53)

Note: Time point is the point of time which represents the difference between the visit time and the time (start point time or 0 time point) when the subjects originally failing the test of the three items above. At the start point time, the mean and standard deviation of physiological age of subjects included in the items “Walk backward”, “Stand from supine position”, “Hop with one foot at least twice” were 25.92 ± 1.99, 26.19 ± 2.21, and 26.51 ± 2.47, respectively.

**Table 6:** Analysis of factors influencing the pass rate of the three items based on Cox regression model.

	Walk Backward (N = 307)		Stand up from Supine Position (N = 371)		Hop with One Foot at Least Twice (N = 405)	
	β Coefficient (OR, 95% CI)	*p* Value	β Coefficient (OR, 95% CI)	*p* Value	β Coefficient (OR, 95% CI)	*p* Value
Child’s physiological age at first measurement	0.15 (1.17, 1.09~1.25)	<0.001	0.36 (1.43, 1.31~1.57)	<0.001	0.37 (1.45, 1.31~1.61)	<0.001
Paternal age						
≤30 yrs	Ref				Ref	
>30 yrs	−0.45 (0.64, 0.48~0.85)	0.002			−0.67 (0.51, 0.32~0.82)	0.005
Region						
Beijing	Ref		Ref		Ref	
Shanghai	0.33 (1.40, 0.90~2.17)	0.14	−0.66 (0.52, 0.31~0.86)	0.01	1.72 (5.61, 1.35~23.23)	0.02
Guangdong	0.65 (1.91, 1.03~3.56)	0.04	−0.21 (0.81, 0.42~1.59)	0.55	1.97 (7.17, 1.51~34.08)	0.01
Paternal education						
bachelor’s degree or above			Ref			
Below bachelor’s degree			−0.48 (0.62, 0.38~1.00)	0.05		

## Abbreviations

C-LAP	Chinese Learning Accomplishment Profile
ASD	Autism Spectrum Disorder
CP	Cerebral Palsy
ATNR	Asymmetrical Tonic Neck Reflex
DS	Down Syndrome
RMST	Restricted Mean Survival Time
KTK	Körperkoordinationstest für Kinder
DD	Development Delay
